# 5-(Sulfamoyl)thien-2-yl 1,3-oxazole inhibitors of carbonic anhydrase II with hydrophilic periphery

**DOI:** 10.1080/14756366.2022.2056733

**Published:** 2022-03-30

**Authors:** Stanislav Kalinin, Alexander Kovalenko, Annika Valtari, Alessio Nocentini, Maxim Gureev, Arto Urtti, Mikhail Korsakov, Claudiu T. Supuran, Mikhail Krasavin

**Affiliations:** a Institute of Chemistry, Saint Petersburg State University, St. Petersburg, Russian Federation; b School of Pharmacy, University of Eastern Finland, Kuopio, Finland; c Department of Neurofarba, Universita degli Studi di Firenze, Florence, Italy; d Digital Biodesign and Personalized Healthcare Research Center, Sechenov First Moscow State Medical University, Moscow, Russian Federation; e Drug Research Program, Division of Pharmaceutical Biosciences, Faculty of Pharmacy, University of Helsinki, Helsinki, Finland; f Pharmaceutical Technology Transfer Center, Ushinsky Yaroslavl State Pedagogical University, Yaroslavl, Russian Federation; g Immanuel Kant Baltic Federal University, Kaliningrad, Russian Federation

**Keywords:** Glaucoma, intraocular pressure, hydrophilicity, bioconjugation, intraocular delivery

## Abstract

Hydrophilic derivatives of an earlier described series of carbonic anhydrase inhibitors have been designed, prepared and profiled against a panel of carbonic anhydrase isoforms, including the glaucoma-related *h*CA II. For all hydrophilic derivatives, computational prediction of intraocular permeability routes showed the predominance of conjunctival rather than corneal absorption. The potentially reactive primary or secondary amine periphery of these compounds makes them suitable candidates for bioconjugation to polymeric drug carriers. As was shown previously, the most active *h*CA II inhibitor is efficacious in alleviating intraocular pressure in normotensive rabbits with efficacy matching that of dorzolamide.

## Graphical Abstract

**Figure:**
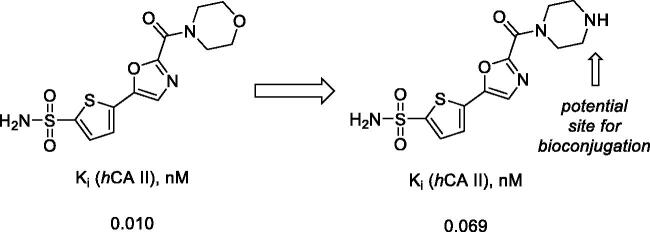

## Introduction

**Figure 1.**
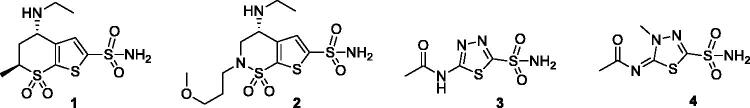
Clinically used antiglaucoma carbonic anhydrase inhibitors.

Glaucoma-related high intraocular pressure can be alleviated by the use of eye drops of prostaglandin analogues, beta blocking agents and carbonic anhydrase inhibitors (CAIs)^1^. The recent approval of rho kinase inhibitors and NO donors significantly expands the range of treatment options^2^^,^^3^. The clinically used topical CAIs for glaucoma treatment include dorzolamide (**1**) and brinzolamide (**2**), compounds that are (a) relatively lipophilic and (b) non-selective as inhibitors of a particular carbonic anhydrase isoform^4^. Acetazolamide (**3**) and methazolamide (**4**) are also used as anti-glaucoma agents (Figure 1), but they are oral medications which frequently cause adverse drug reactions^5^. Potent and selective inhibition of carbonic anhydrase II isoform (*h*CA II) is an important mechanism of action due to the critical importance of this enzyme in reduction of glaucoma-related intraocular pressure^6^.

Topical ocular drugs are typically designed as rather lipophilic, because they absorb to the eye across the cornea^7^. Lipophilicity leads to decreased water solubility and, thus, lowers the achievable drug concentration in the tear fluid. On the contrary, higher concentration in the tear fluid can be achieved with hydrophilic compounds. Such compounds may absorb into their ocular targets via conjunctiva and sclera that allow permeation of relatively hydrophilic compounds^8^. Specifically designing hydrophilic compounds that can utilise this route will lower the loss of hydrophilic compounds to the blood stream across conjunctiva^8^. Anti-glaucoma CAIs exert their action in the ciliary body located next to sclera, thereby making non-corneal absorption of highly potent, hydrophilic derivatives an interesting approach. Moreover, in comparison to the cornea, the conjunctiva has wider inter-cellular space for permeation of hydrophilic compounds^9^.

**Figure 2.**
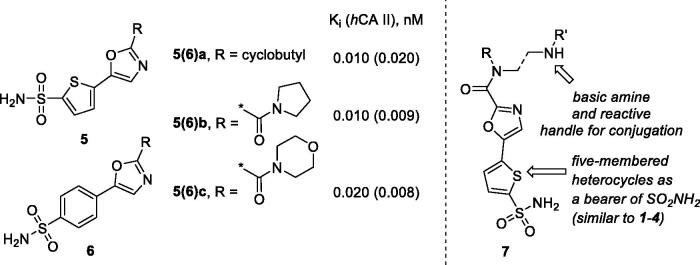
Earlier reported potent *h*CAII inhibitors **5(6)a–c** and their modified hydrophilic thiophene analogues **7** designed and investigated in this work.

Previously, we described a series of 5-(sulfamoyl)thien-2-yl 1,3-oxazoles **5a–c** which displayed a remarkably potent inhibition profile towards human carbonic anhydrase (CA, EC 4.2.1.1) and, in particular, its *h*CA II isoform^10^ which is the primary target for intraocular pressure-reducing antiglaucoma drugs.^6^ Later on, a related – and similarly potent against *h*CA II – benzenesulfonamide series (**6a–c**) showed high efficacy *in vivo* lowering ocular hypertension in rabbits. Furthermore, the high potency and the pronounced selectivity towards the CA isoform of this series was rationalised by X-ray crystallographic structure of complex of **6c** with the protein^11^. Considering that compounds **5a–c** contain the primary sulphonamide group linked to a thiophene moiety, it makes them structurally closer to the clinically used drugs **1**–**4** all of which have a five-membered heterocyclic core as a primary sulphonamide-bearing scaffold. Thus, we selected carboxamides **5b–c** as the prototype scaffold for the introduction of peripheral functional groups which would increase the resulting compounds’ hydrophilicity and also a reactive ‘handle’ for subsequent chemical conjugation to polymer nanoparticles. These notions resulted in the design of series **7** (Figure 2).

Eye drop treatment for glaucoma is notoriously hampered by the poor patient compliance and the progression of the disease and loss of vision^12^. Longer-acting intraocular drug delivery with polymeric systems could potentially solve this issue^13^. New compounds **7** were designed with this downstream goal in mind, since their structure could allow conjugation to the polymeric carriers *via* amide and other potentially biodegradable linkages. On the other hand, the inherent hydrophilicity of these compounds was seen as potentially beneficial as hydrophilic compounds, even when liberated from a polymer carrier, display slower clearance from the intraocular spaces^14^. Thus, even with similar on-target potencies, more hydrophilic drugs, once delivered to the intraocular space, are expected to have a lower clearance and would require smaller dose per day to exert their actions. Even taken alone, more hydrophilic *h*CA II inhibitors will have potential as traditional eye drop medications if they could be potentially delivered across the conjunctiva-sclera route to the ciliary body.

As cautioned earlier^15^, ‘decorating’ a more lipophilic potent *h*CA II inhibitor with outright hydrophilic moieties (i.e. moving from **5** to **7**) carries a potential risk of losing the desired *h*CA II potency. As one must bear in mind, the active site of carbonic anhydrase has a very characteristic topology where a hydrophobic half of the protein surface is clearly delineated from the hydrophilic one^16^. Thus, replacing a relatively hydrophopic groups in **5a–c** with a large hydrophilic carboxamide groups could, in principle, deprive **7** of desired affinity to *h*CA II. Despite these potential risks we set off to synthesise a set of compounds **7** for investigation of their carbonic anhydrase inhibitory potency *in vitro* and subsequent efficacy study of the best inhibitor intraocular pressure-lowering agents *in vivo*. Herein, we report the results of these studies.

## Results and discussion

**Scheme 1.**
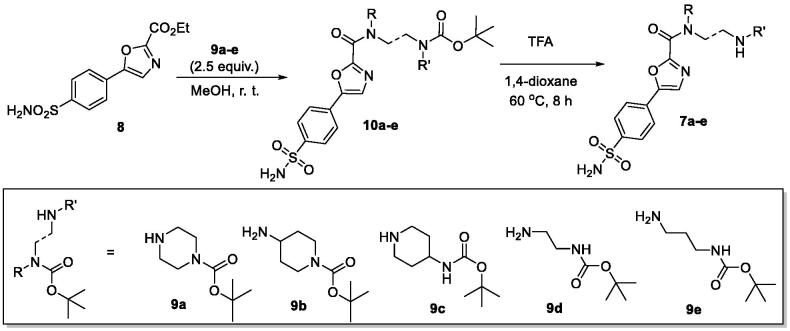
Synthesis of hydrophilic sulphonamides **7a–e** investigated in this work.

The key building block – ethyl 5–(4-sulfamoylphenyl)oxazole-2-carboxylate (**8**) – was synthesised in several straightforward steps from α-aminoacetophenone hydrochloride as described previously^10^^,^^15^. The electron-withdrawing influence of the sulphonamide group on the electrophilicity of the ester functionality in **8** turned out to be of advantage in subsequent synthesis of the target compounds **7a–e**. Indeed, on reaction requiring no additional activation, with 2.5-fold excess of mono-Boc-protected dibasic amines **9a–e** at r.t. in MeOH, respective amides **10a–e** were obtained and deprotected with TFA in 1,4-dioxane at 60 °C and purified chromatographically to give the target compounds **7a–e** (Scheme 1).

The inhibitory profile obtained for sulphonamides **7a–e** in a stopped-flow kinetics assay against human CA I, II, IV and XII is shown in Table 1. In addition to *h*CA II, the other three isoforms were selected to preliminarily gauge the off-target profile of the compounds intended to inhibit the target isoform. Moreover, inhibition profile against *h*CA IV and XII was thought to be of significance as these isoforms are also involved in the secretion of the intraocular liquor^17^.

**Table 1.** Inhibitory activity of compounds **7a–e** against the target (*h*CA II) as well as selected off-target (*h*CA I, IV and XII) isoforms.

Compound	Structure	K_i_ (nM)^a^			
		*h*CA I	*h*CA II	*h*CA IV	*h*CA XII
7a	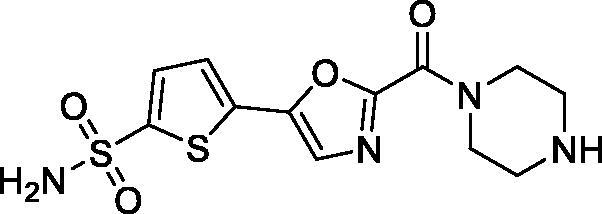	4.0	0.069	21.6	3.9
7b	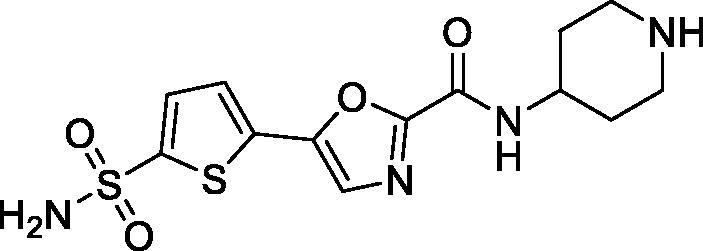	56.8	0.92	23.7	8.9
7c	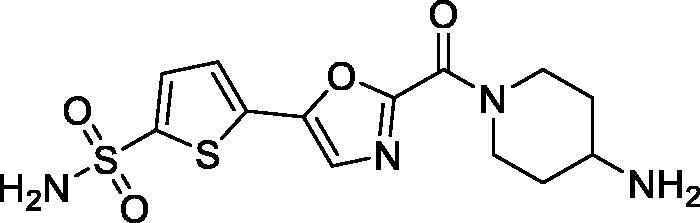	31.3	0.41	30.6	5.7
7d	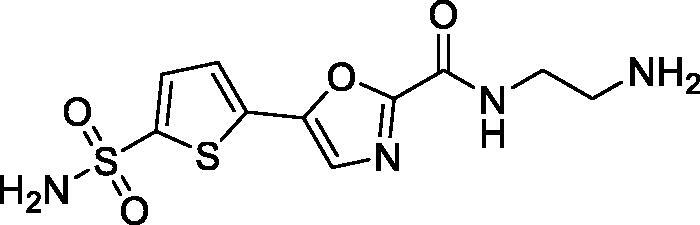	72.9	3.9	5.2	9.3
7e	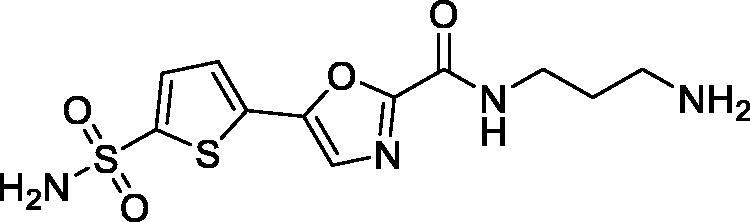	58.3	3.1	4.6	8.8
3^b^		250	12	75	5.7

^a^Mean from three different assays by stopped flow technique (errors were in the range of ± 5–10% of the reported values).

^b^Sulfonamide inhibitor acetazolamide (AAZ) used as a reference pan-CA inhibitor in stopped flow CO_2_ hydrase assay.

To our delight, all four inhibitors **6a–d** preserved the potent inhibition profile against the target *h*CA II isoform (although their *h*CA II potency deteriorated somewhat compared to the less hydrophilic initial leads **5a–c**) and a clearly better *h*CA II selectivity profile compared to acetazolamide (**4**) employed as a reference inhibitor. Interestingly, the replacement of the morpholine oxygen atom in **5c** with hydrogen bond donating/accepting piperazine *NH* in compound **7a** (a rather drastic change from the standpoint of potential molecular interactions which resulted in the change of the binding mode, *vide infra*) led to only a three-fold drop in *h*CA II potency. This clearly makes compound **7a** stand out as the hydrophilic (and potentially ‘bioconjugatable’) follow-on to compound **5c**. Of course, the ultimate efficacy profile of this inhibitor reducing the glaucoma-related intraocular pressure (IOP) would depend on a multitude of factors among which permeability characteristics (intrinsically linked to a favourable set of molecular parameters) will be of significance.

**Figure 3.**
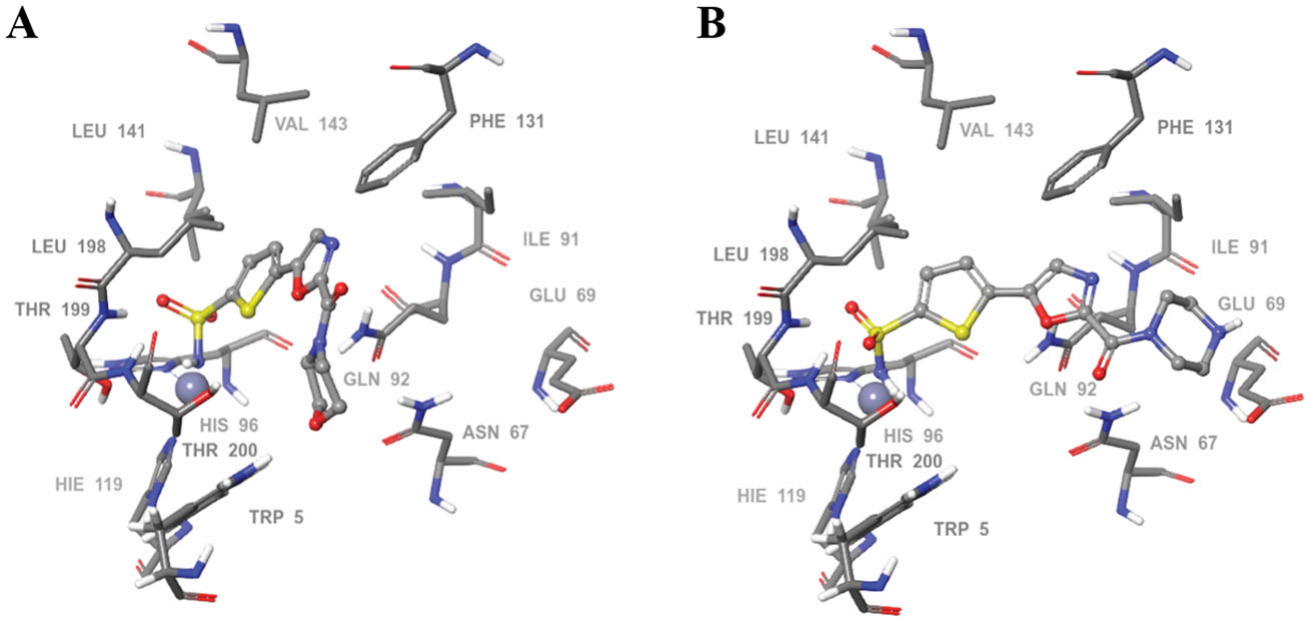
Binding poses of **5c** (A) and **7a** (B) in the *h*CAII active site.

In order to visualise the binding of the prototype compound **5c** in comparison to the hydrophilic lead derivative **7a** and to possibly understand the origins of the essentially preserved *h*CA II potency in case of the latter, we performed the docking of both ligands into the active site *h*CA II. In the case of both prototype molecule **5c** and the advanced hydrophilic lead compound **7a** the thiophene sulphonamide moiety, predictably, acted as a zinc binding group displaying typical orientation which is well known from a wide range of crystallographic studies^18^. Specifically, the sulphonamide moiety interacted with the catalytic Zn^2+^ ion as well as with Thr199. At the same time, the thiophene ring was oriented towards the hydrophobic pocket lined up with the residues Leu141, Val143, and Phe131. Furthermore, the 1,3-oxazole ring of the ligands was involved in interactions with Phe131 and formed a hydrogen bond with Gln92. Interestingly, we found the morpholineamide moiety in the compound **5c** was oriented towards the NH-groups of the Trp5 and Asn67. In contrast, the piperazine ring in compound **7a** formed a salt bridge with Glu69. As it follows from this analysis, presumably, the ligand–protein interactions displayed by both morpholineamide moiety in **5a** and piperazine amide substituent in **7a** resulted in the favourable energy for the molecules’ binding within the active site of *h*CA II and thus leading the potent inhibitory action of the compounds against the CA isoform (Figure 3).

**Figure 4.**
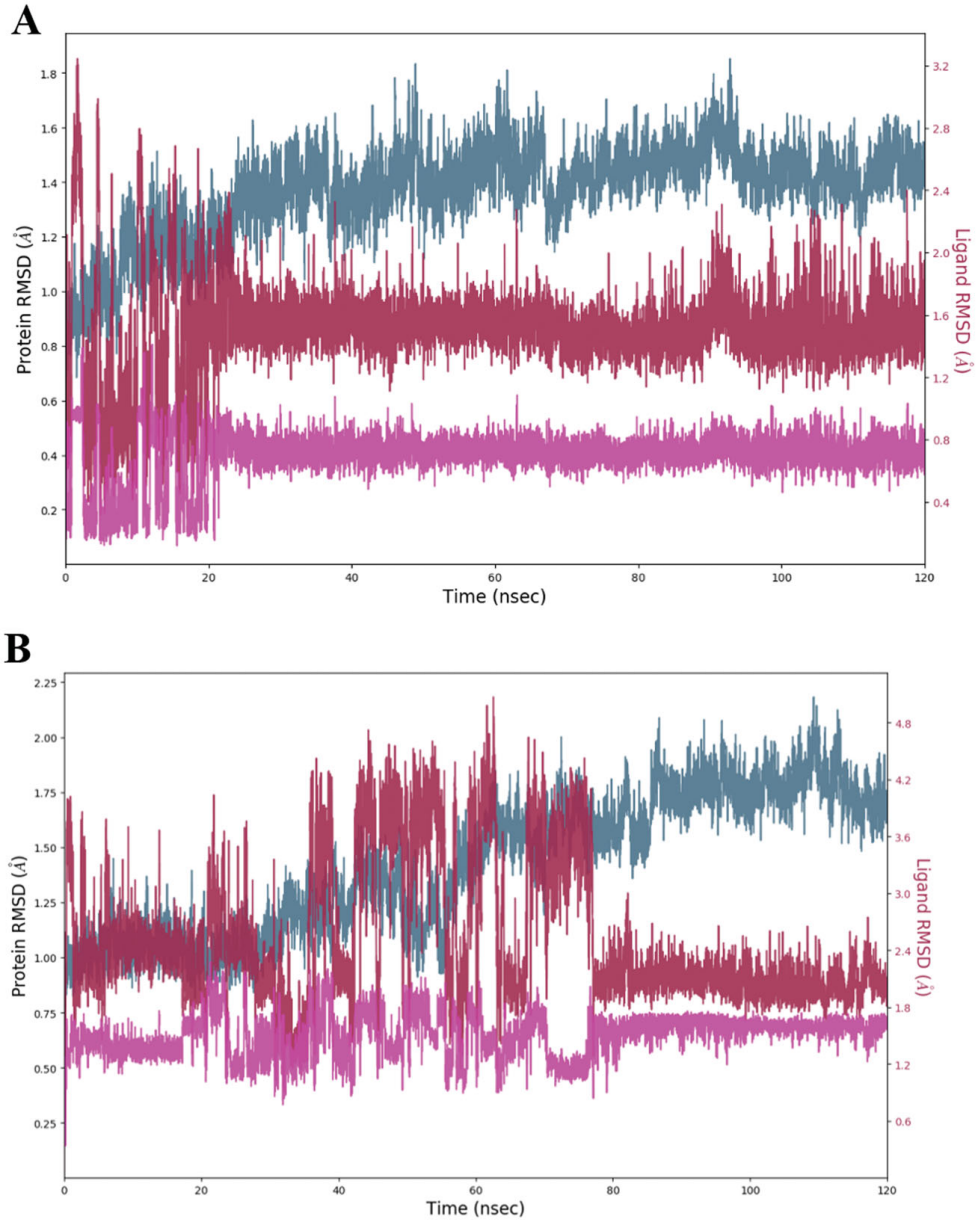
RMSD changes observed for the complexes ‘acetazolamide – *h*CA II’ (A) and ‘compound **7a** – *h*CA II’ (B) during a 120 ns molecular dynamics simulation.

In order to test the robustness of the docking poses identified, we performed 120 ns molecular dynamics simulation of ligand **7a** docked in the active site of *h*CA II in comparison with the clinically used (non-selective) *h*CA II inhibitor acetazolamide (**3**). The RMSD values of the protein backbone (blue), the ligand relative to *h*CA II (red) and the ligand relative to its original, pre-simulation docking pose (purple) were found to stabilise to fit the range of 1–3 Å (robust fit) within 23.36 ns for acetazolamide and within 77 ns for ligand **7a** (Figure 4). The longer relaxation time observed for **7a** has likely to do with the greater conformational flexibility of the piperazine carboxamide side chain which took longer to restore the network of critical hydrogen-bonding contacts. Overall, the molecular dynamics simulation demonstrated the robustness of the docking pose presented in Figure 3(B).

**Figure 5.**
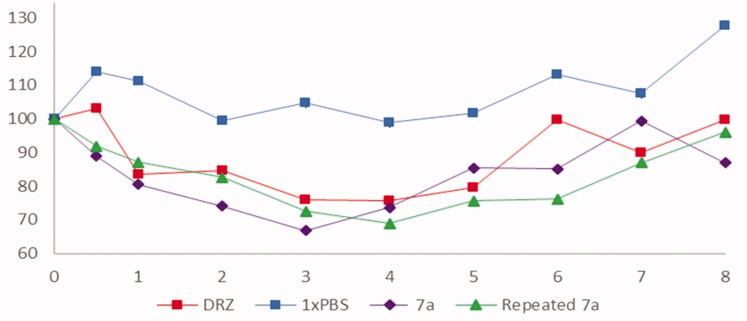
Percentage change in IOP (*y* axis) over time (*x* axis) after administration of compound **7a** (two independent experiments), negative control phosphate buffered saline (PBS) and positive control dorzolamide (DRZ) in albino rabbits (*n* = 6).

The intraocular pressure (IOP) lowering effect of newly developed hydrophilic *h*CAII inhibitor **7a** was tested in normotensive New Zealand White rabbits^19^. The results are shown as percentage changes in Figure 5. Compound **7a** (1% eye drop) (tested twice consecutively) showed a clear IOP lowering effect which was comparable to the effect produced by **1** (dorzolamide, administered as 2% eye drops).

For compounds **7a–e**, we have calculated a series of chemical descriptors (Table 2) from which critical ocular permeability parameters can be deduced. It is apparent, that all five compounds are distinctly hydrophilic.

**Table 2.** Chemical descriptors of carbonic anhydrase inhibitors **7a–e** (calculated using ACDLabs 12.0).^a^

Compound	HBa	HBd	HBtot	LogP	MW	LogD_8.0_	PSA	logPSA
7a	3	11	−0.42	0.000861	342.39	0.38	155.15	2.191
7b	4	12	−1.46	−0.93	356.42	0.84	163.94	2.215
7c	4	12	−1.24	−0.68	356.42	0.42	169.14	2.228
7d	5	13	−1.11	−0.60	316.36	0.10	177.93	2.250
7e	5	13	−1.84	−1.30	330.38	0.36	177.93	2.250

^a^MW: molecular weight; HBa: hydrogen bond acceptors; HBd: hydrogen bond donors; HBtot: total amount of hydrogen bond formers; LogP: logarithmic value of partition coefficient; LogD_7.4_/LogD_8.0_: logarithmic value of distribution coefficient at pH 7.4/8.0; PSA: polar surface area; LogPSA: logarithmic value of polar surface area.

The chemical descriptors presented in Table 2 allowed us to calculate the predicted corneal and conjunctival permeability values for compounds **7a–e** in comparison with dorzolamide (**1**) (Table 3). These calculations are based on the earlier formulas by Kidron et al.^20^ and Ramsay et al.^21^^,^^22^. It is apparent that the conjunctival permeation route becomes a principal one for hydrophilic compounds **7a–e** in comparison with more lipophilic dorzolamide (**1**) (Table 4).

**Table 3.** Calculated permeability (*P*_app_) values of dorzolamide (**1**) and compounds **7a–e**.

Compound	Cornea (rabbit)		Cornea (porcine)		Conjunctiva (porcine)	
	*P*_app_ (cm/s)	% of 1	*P*_app_ (cm/s)	% of 1	*P*_app_ (cm/s)	% of 1
1	7.79E − 06	100	1.75E − 07	100	1.86E − 06	100
7a	9.68E − 07	12	1.71E − 07	98	1.83E − 06	98
7b	3.27E − 07	4	1.20E − 07	69	1.47E − 06	79
7c	3.76E − 07	5	1.18E − 07	67	1.45E − 06	78
7d	2.68E − 07	3	8.29E − 08	48	1.17E − 06	63
7e	1.68E − 07	2	8.29E − 08	48	1.17E − 06	63

**Table 4.** Formulas for estimating permeability properties of carbonic anhydrase inhibitors.

	Formula	References
Corneal permeability of rabbit (cm/s)	LogPapp = −3.885 − 0.183(HBtot)+0.277(logD7.4)	19
Corneal permeability of porcine (cm/s)	LogPapp = −4.6823 − 0.7670(logPSA)−0.1346 (HBd)+3.0024(Halogen ratio)	20
Conjunctival permeability of porcine (cm/s)	LogPapp = −4.1594 − 0.6121(logPSA)- 0.0792(HBd)+3.2914(Halogen ratio)	21

LogPapp: logarithmic value of apparent permeability; HBtot: total amount of hydrogen bond formers; LogD7.4: logarithmic value of distribution coefficient at pH 7.4; LogPSA: logarithmic value of polar surface area; HBd: hydrogen bond donors; Halogen ratio: sum of all halogens divided by the sum of all heavy atoms excluding hydrogen.

In summary, we have described next-generation 5-(sulfamoyl)thien-2-yl 1,3-oxazole carbonic anhydrase inhibitors endowed with a primary or secondary amine periphery. The compounds were designed with a dual goal of increasing compounds’ hydrophilicity and provide a reactive ‘handle’ for potential conjugation to sustained-release nanoparticles. Increased hydrophilicity, while desirable for increased drug residence in the intraocular space could be generally viewed as an obstacle for corneal drug absorption. However, hydrophilic compounds may be efficiently absorbed *via* conjunctiva and thus have greater efficacy which may be expected if corneal absorption alone is considered. Out of the compounds described herein, the lead compound (**7a**) displayed a potent and selective inhibition of *h*CA II isoform, a glaucoma target and showed comparable efficacy as 1% eye drops in reducing the intraocular pressure in normotensive rabbit to that of clinically used 2% dorzolamide eye drops. This is despite the fact that the corneal permeability of these hydrophilic compounds was predicted to be significantly lower than that of dorzolamide. The data additionally support the concept of hydrophilic compounds permeating across the conjunctiva and sclera into the ciliary body.

## Supplementary Material

Supplemental Material

## References

[1] Jonas JB, Aung T, Bourne RR, et al. Glaucoma. Lancet 2017;390:2183–93. 28577860 10.1016/S0140-6736(17)31469-1

[2] Mincione F, Nocentini A, Supuran CT. Advances in the discovery of novel agents for the treatment of glaucoma. Expert Opin Drug Discov 2021;16:1209–25. 33914670 10.1080/17460441.2021.1922384

[3] Guglielmi P, Carradori S, Campestre C, Poce G. Novel therapies for glaucoma: a patent review (2013–2019). Expert Opin Ther Pat 2019;29:769–80. 31385719 10.1080/13543776.2019.1653279

[4] Schmidl D, Schmetterer L, Garhöfer G, Popa-Cherecheanu A. Pharmacotherapy of glaucoma. J Ocul Pharmacol Ther 2015;31:63–77. 25587905 10.1089/jop.2014.0067 PMC4346603

[5] Kanner E, Tsai JC. Glaucoma medications. Drugs Aging 2006;23:321–32. 16732691 10.2165/00002512-200623040-00005

[6] a) Maren TH. Carbonic anhydrase: chemistry, physiology, and inhibition. Physiol Rev 1967;47:595–781. 4964060 10.1152/physrev.1967.47.4.595

[7] Maurice DM, Mishima S. Ocular pharmacology. In: Sears M, ed. Handbook of experimental pharmacology. Berlin-Heidelberg: Springer-Verlag; 1984:16–19.

[8] Ahmed I, Patton TF. Importance of the noncorneal absorption route in topical ophthalmic drug delivery. Invest Ophthalmol Vis Sci 1985;26:584–7. 3884542

[9] Hämäläinen KM, Kananen K, Auriola S, et al. Characterization of paracellular and aqueous penetration routes in cornea, conjunctiva, and sclera. Invest Ophthalmol Vis Sci 1997;38:627–34. 9071216

[10] Krasavin M, Korsakov M, Dorogov M, et al. Probing the 'bipolar' nature of the carbonic anhydrase active site: aromatic sulfonamides containing 1,3-oxazol-5-yl moiety as picomolar inhibitors of cytosolic CA I and CA II isoforms. Eur J Med Chem 2015;101:334–47. 26160114 10.1016/j.ejmech.2015.06.022

[11] Ferraroni M, Lucarini L, Masini E, et al. 1,3-Oxazole-based selective picomolar inhibitors of cytosolic human carbonic anhydrase II alleviate ocular hypertension in rabbits: potency is supported by X-ray crystallography of two leads. Bioorg Med Chem 2017;25:4560–5. 28728897 10.1016/j.bmc.2017.06.054

[12] Broadway DS, Cate H. Pharmacotherapy and adherence issues in treating elderly patients with glaucoma. Drugs Aging 2015;32:569–81. 26136215 10.1007/s40266-015-0282-9

[13] Yadav KS, Rajpurohit R, Sharma S. Glaucoma: current treatment and impact of advanced drug delivery systems. Life Sci 2019;221:362–91. 30797820 10.1016/j.lfs.2019.02.029

[14] Del Amo EM, Rimpelä A-K, Heikkinen E, et al. Pharmacokinetic aspects of retinal drug delivery. *Prog Retin Eye Res 2017;57:134–85. 28028001 10.1016/j.preteyeres.2016.12.001

[15] Kalinin S, Valtari A, Ruponen M, et al. Highly hydrophilic 1,3-oxazol-5-yl benzenesulfonamide inhibitors of carbonic anhydrase II for reduction of glaucoma-related intraocular pressure. Bioorg Med Chem 2019;27:115086. 31515057 10.1016/j.bmc.2019.115086

[16] Nocentini A, Angeli A, Carta F, et al. Reconsidering anion inhibitors in the general context of drug design studies of modulators of activity of the classical enzyme carbonic anhydrase. J Enzyme Inhib Med Chem 2021;36:561–80. 33615947 10.1080/14756366.2021.1882453 PMC7901698

[17] Scozzafava A, Supuran CT. Glaucoma and the applications of carbonic anhydrase inhibitors. Subcell Biochem 2014;75:349–59. 24146387 10.1007/978-94-007-7359-2_17

[18] Lomelino CL, Andring JT, McKenna R. Crystallography and its impact on carbonic anhydrase research. Int J Med Chem 2018;2018:9419521. 30302289 10.1155/2018/9419521 PMC6158936

[19] Pilipenko I, Korzhikov-Vlakh V, Valtari A, et al. Mucoadhesive properties of nanogels based on stimuli-sensitive glycosaminoglycan-graft-pNIPAAm copolymers. Int J Biol Macromol 2021;186:864–72. 34274401 10.1016/j.ijbiomac.2021.07.070

[20] Kidron H, Vellonen K-S, Amo E, et al. Prediction of the corneal permeability of drug-like compounds. Pharm Res 2010;27:1398–407. 20387098 10.1007/s11095-010-0132-8

[21] Ramsay E, Del Amo E, Toropainen E, et al. Corneal and conjunctival drug permeability: systematic comparison and pharmacokinetic impact in the eye. Eur J Pharm Sci 2018;119:83–9. 29625211 10.1016/j.ejps.2018.03.034

[22] Ramsay E, Ruponen M, Picardat T, et al. Impact of chemical structure on conjunctival drug permeability: adopting porcine conjunctiva and cassette dosing for construction of *in silico* model. J Pharm Sci 2017;106:2463–71. 28479360 10.1016/j.xphs.2017.04.061

