# Supplementary material for: 5-(Sulfamoyl)thien-2-yl 1,3-oxazole inhibitors of carbonic anhydrase II with hydrophilic periphery
Source: J Enzyme Inhib Med Chem. 2022 Mar 30;37(1):1005–11. doi: 10.1080/14756366.2022.2056733 (PMC8973362; doi:10.1080/14756366.2022.2056733)
Supplement: Supplemental Material [file IENZ_A_2056733_SM4846.pdf]

Supporting information for

5-(Sulfamoyl)thien-2-yl 1,3-Oxazole Inhibitors of Carbonic Anhydrase II with Hydrophilic Periphery

Stanislav Kalinin, Alexander Kovalenko, Annika Valtari, Alessio Nocentini, Maxim Gureev, Arto Urtti*, Mikhail Korsakov, Claudiu T. Supuran* and Mikhail Krasavin*

Contents

Synthetic procedures towards compounds **7a-e** pp. 2 – 3

Copies of ^{1}H and ^{13}C NMR spectra for compounds **7a-e** pp. 4 – 9

Carbonic anhydrase inhibition assay p. 9

Docking and molecular dynamics studies p. 9

Intraocular pressure studies p. 9

References p. 10

Synthetic procedures towards compounds **7a-e**

General procedure 1 (GP1) for the preparation of compounds 7a–e

To a solution of **8** (0.20 g, 0.66 mmol) in methanol corresponding mono-*Boc*-protected diamine (1.32 mmol) was added and the mixture was refluxed for 12 hours. The solution was then removed by rotary evaporation, the residue was dissolved in chloroform (5 mL), washed by 10% solution of HCl (pH 3.5) (2x2.5 mL) and H_2O (3x2.5mL), dried over Na_2SO_4 and concentrated. The residue was loaded into a test tube and dissolved in dry 1,4-dioxane (4 mL). 4M HCl solution in 1,4-dioxane (1 mL) was added and the reaction mixture was stirred for 8 hours at 60°C. White precipitate formed was filtered and, if needed, were purified by HPLC using acetonitrile:H_2O:trifluoroacetic acid system.

4-(5-(5-sulfamoylthiophen-2-yl)oxazole-2-carbonyl)piperazin-1-ium 2,2,2-trifluoroacetate (7a)

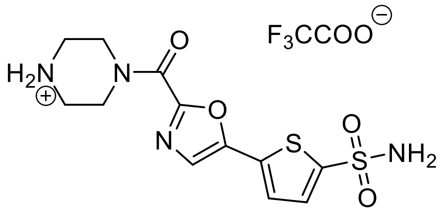

Prepared according to GP1. Yield: 32% ^{1}H NMR (400 MHz, D_2O) δ 7.65 – 7.58 (m, 2H, CH*Thiophene*, CH*Oxazole*), 7.45 (d, *J* = 4.1 Hz, 1H, CH*Thiophene*), 4.70 (s, SO_2NH_2, NH_2^+ in exchange with water, 4H), 4.44 (t, *J* = 5.4 Hz, 2H, CH_2), 4.01 (t, *J* = 5.4 Hz, 2H, CH_2), 3.42 (t, *J* = 5.4 Hz, 4H, CH_2) ppm. ^{13}C NMR (100 MHz, D_2O) δ 156.0, 152.6, 147.0, 143.2, 133.5, 132.3, 126.5, 124.3, 43.8, 43.2, 42.8, 39.8 ppm. HRMS (ESI), m/z calcd for $C_{12}H_{15}N_4O_4S_2^+$ 343.0535 $[M+H]^+$ found 343.0522.

4-(5-(5-sulfamoylthiophen-2-yl)oxazole-2-carboxamido)piperidin-1-ium 2,2,2-trifluoroacetate (7b)

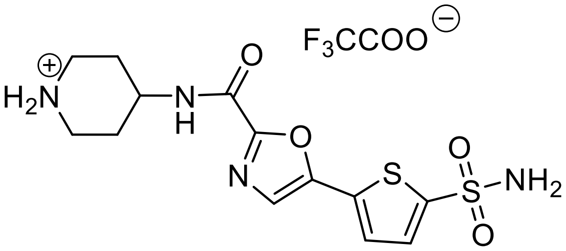

Prepared according to GP1. Yield: 54% ^{1}H NMR (400 MHz, D_2O) δ 7.54 (d, *J* = 4.0 Hz, 1H, CH*Thiophene*), 7.49 (s, 1H, CH*Oxazole*), 7.34 (d, *J* = 4.0 Hz, 1H, CH*Thiophene*), 4.70 (s, SO_2NH_2, NH_2^+ in exchange with water, 4H), 4.18 – 4.06 (m, 1H, CH), 3.55 – 3.47 (m, 2H, CH_2), 3.21 – 3.09 (m, 2H, CH_2), 2.26 – 2.18 (m, 2H, CH_2), 1.94 – 1.81 (m, 2H, CH_2) ppm. ^{13}C NMR (126 MHz, DMSO-d_6) δ 154.5, 154.4, 147.1, 146.7, 132.3, 131.4, 126.5, 125.3, 45.0, 42.7 (2C), 28.4 (2C) ppm. HRMS (ESI), m/z calcd for $C_{13}H_{17}N_4O_4S_2^+$ 357,0691 $[M+H]^+$ found 357,0679.

1-(5-(5-sulfamoylthiophen-2-yl)oxazole-2-carbonyl)piperidin-4-aminium hydrochloride (7c)

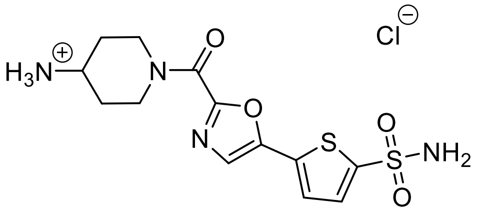

Prepared according to GP1. Yield: 64% ^{1}H NMR (400 MHz, D_2O) δ 7.64 (d, J = 4.0 Hz, 1H, $CH_{Thiophene}$), 7.61 (s, 1H, $CH_{Oxazole}$), 7.47 (d, J = 4.0 Hz, 1H, $CH_{Thiophene}$), 4.84 – 4.76 (m, 1H, CH_2), 4.70 (s, SO_2NH_2, NH_3^+ in exchange with water, 5H), 4.62 – 4.53 (m, 1H, CH_2), 3.62 – 3.50 (m, 1H, CH), 3.44 – 3.32 (m, 1H, CH_2), 3.10 – 2.98 (m, 1H, CH_2), 2.25 – 2.11 (m, 2H, CH_2), 1.81 – 1.61 (m, 2H, CH_2) ppm. ^{13}C NMR (100 MHz, D_2O) δ 156.2, 153.2, 146.7, 143.0, 133.6, 132.4, 126.3, 124.0, 47.8, 45.3, 41.6, 29.9, 29.0 ppm. HRMS (ESI), m/z calcd for $C_{13}H_{17}N_4O_4S_2^+$ 357,0691 $[M+H]^+$ found 357,0701.

2-(5-(5-Sulfamoylthiophen-2-yl)oxazole-2-carboxamido)ethan-1-aminium 2,2,2-trifluoroacetate (7d)

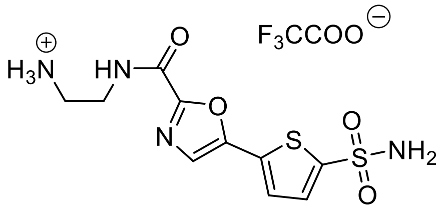

Prepared according to GP1. Yield: 51% ^{1}H NMR (400 MHz, DMSO-d_6, as TFA salt) δ 9.10 (t, J = 5.9 Hz, 1H, COONHCH), 7.96 (s, 1H, $CH_{Oxazole}$), 7.89 (s, 2H, SO_2NH_2), 7.83 (s, 3H, NH_3^+), 7.62 (d, J = 3.9 Hz, 1H, $CH_{Thiophene}$), 7.61 (d, J = 3.9 Hz, 1H, $CH_{Thiophene}$), 3.53 (q, J = 6.1 Hz, 2H, CH_2), 3.01 (h, J = 6.0 Hz, 2H, CH_2) ppm. ^{13}C NMR (100 MHz, DMSO, as TFA salt, the signal of TFA was not included) δ 155.4, 154.5, 147.1, 146.8, 132.2, 131.4, 126.5, 125.4, 39.0, 37.3 ppm. HRMS (ESI), m/z calcd for $C_{10}H_{13}N_4O_4S_2^+$ 317.0378 $[M+H]^+$ found 317.0367.

3-(5-(5-sulfamoylthiophen-2-yl)oxazole-2-carboxamido)propan-1-aminium 2,2,2-trifluoroacetate (7e)

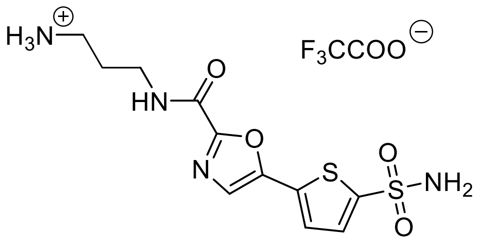

Prepared according to GP1. Yield: 50% ^{1}H NMR (400 MHz, DMSO-d_6) δ 9.14 (t, J = 6.0 Hz, 1H, COONHCH), 7.93 (s, 1H, $CH_{Oxazole}$), 7.88 (s, 2H, SO_2NH_2), 7.76 (s, 3H, NH_3^+), 7.62 (d, J = 3.9 Hz, 1H, $CH_{Thiophene}$), 7.60 (d, J = 3.9 Hz, 1H, $CH_{Thiophene}$), 3.34 (q, J = 6.6 Hz, 2H, CH_2), 2.92 – 2.78 (m, 2H, CH_2), 1.82 (p, J = 6.9 Hz, 2H, CH_2) ppm. ^{13}C NMR (126 MHz, DMSO, as TFA salt, the signal of TFA was not included) δ 155.05, 154.63, 147.06, 146.73, 132.30, 131.37, 126.44, 125.35, 37.30, 36.67, 27.58 ppm. HRMS (ESI), m/z calcd for $C_{11}H_{15}N_4O_4S_2^+$ 331.0535 $[M+H]^+$ found 331.0528.

Copies of ^{1}H and ^{13}C spectra of compound **7a**

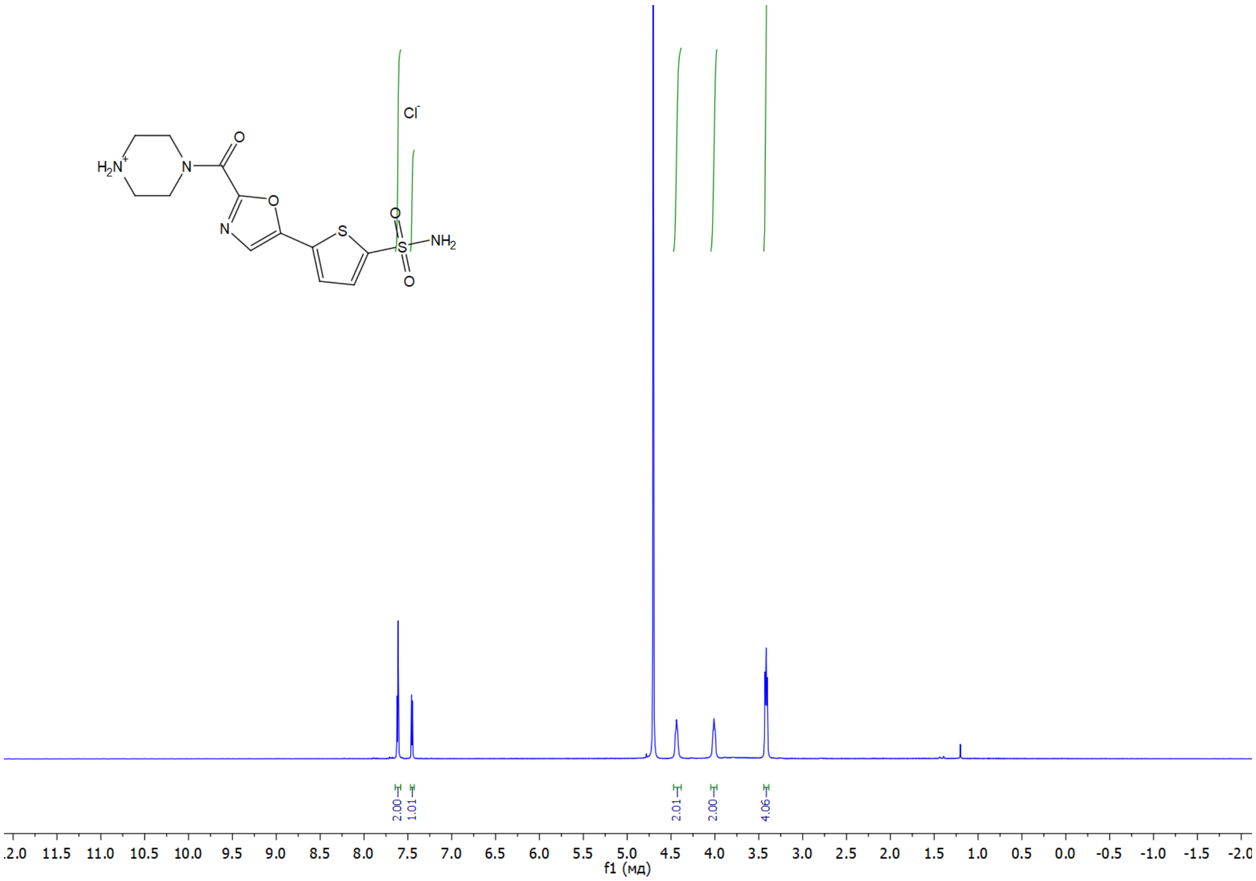

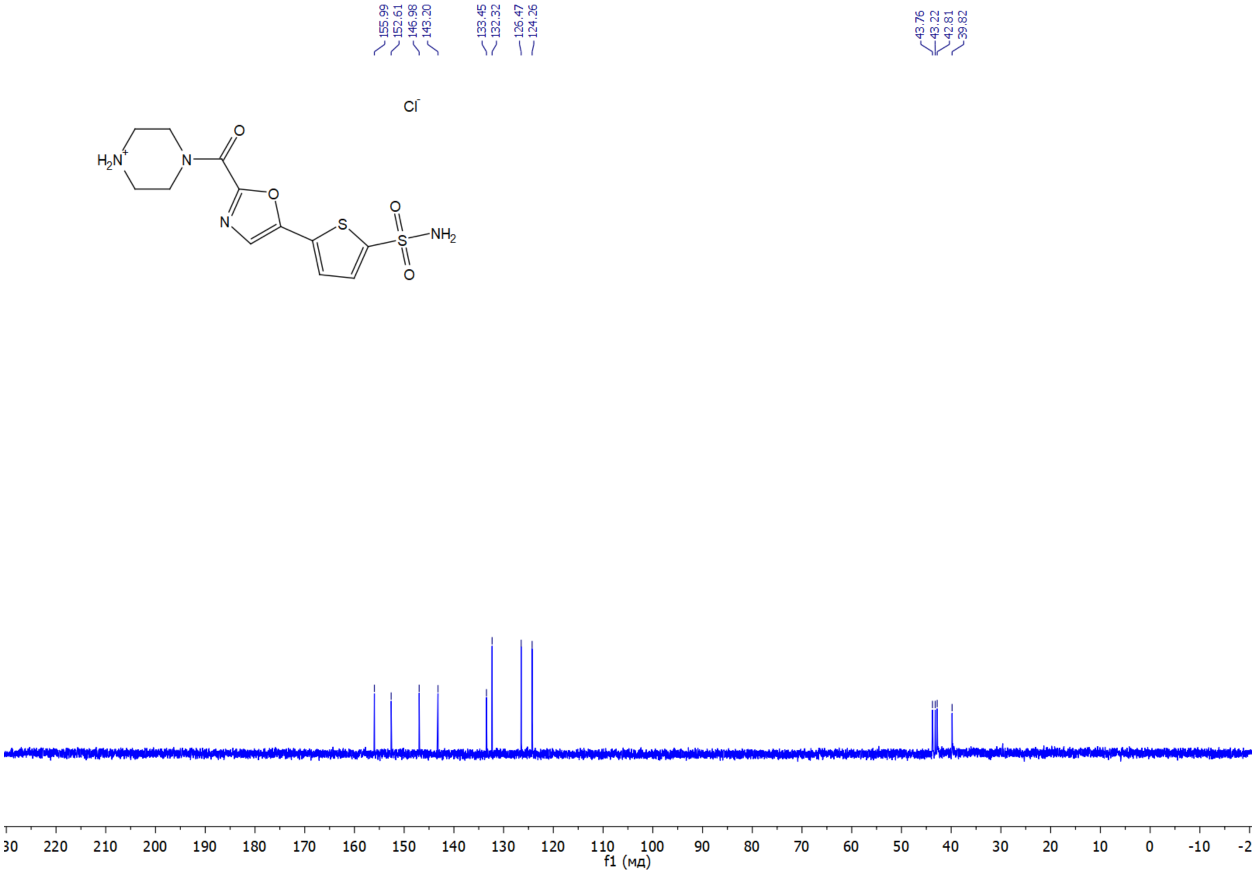

Copies of ^{1}H and ^{13}C spectra of compound **7b**

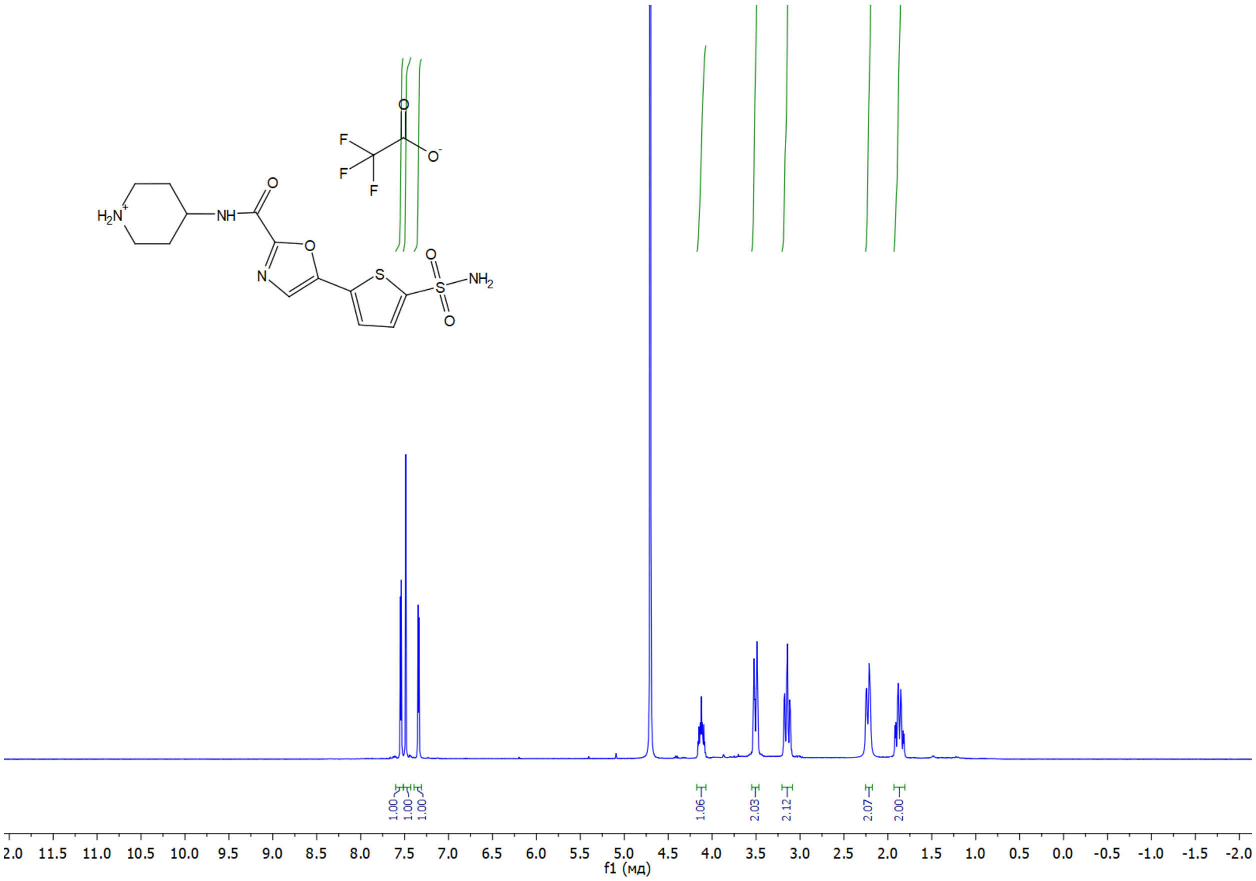

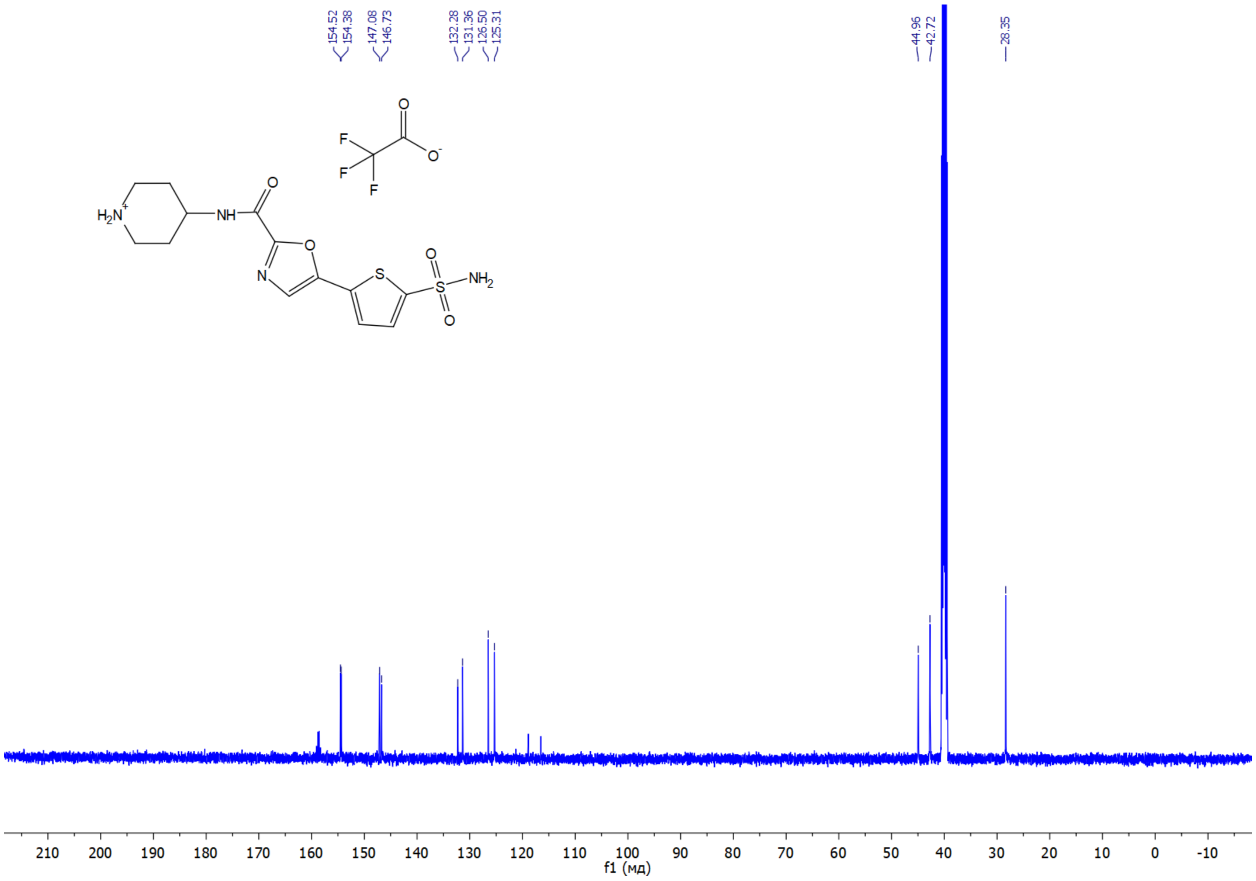

Copies of ^{1}H and ^{13}C spectra of compound **7c**

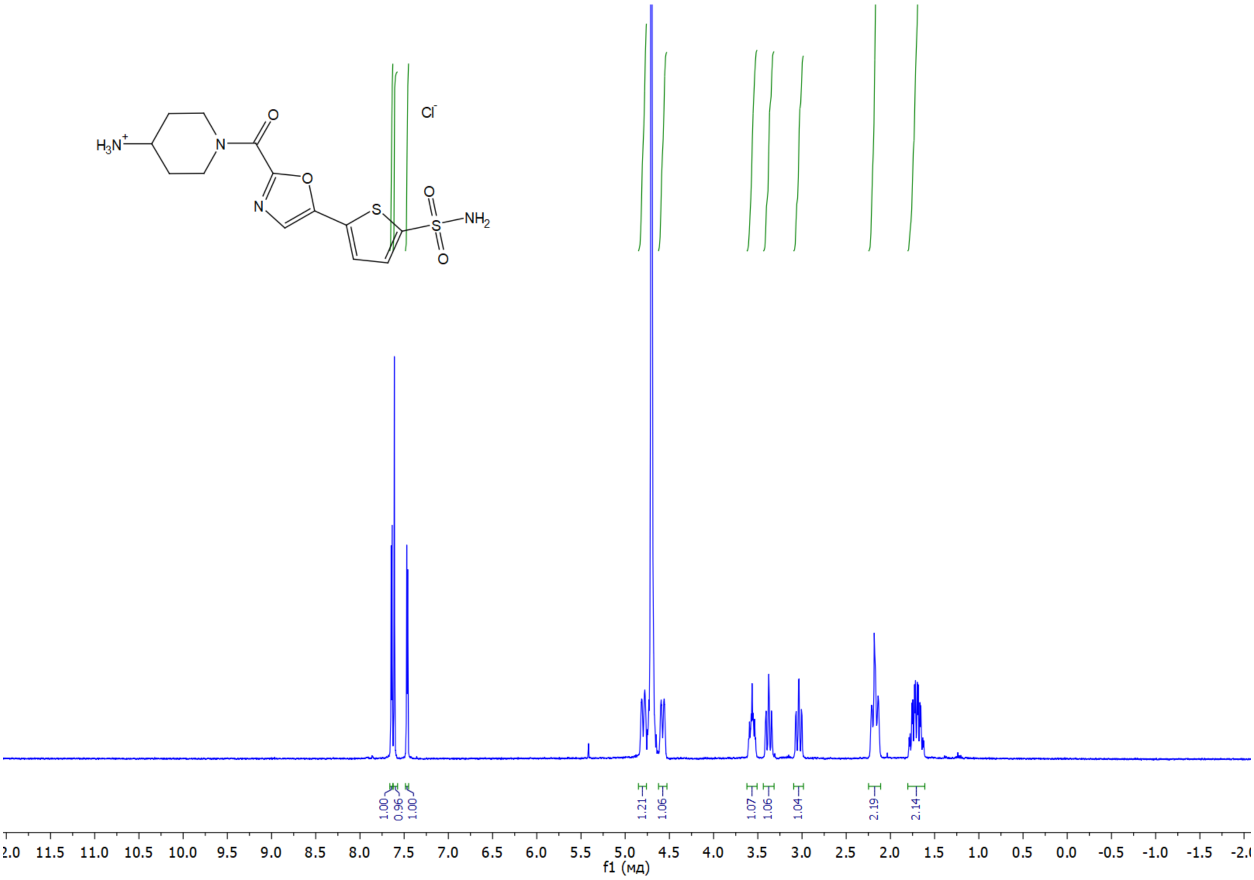

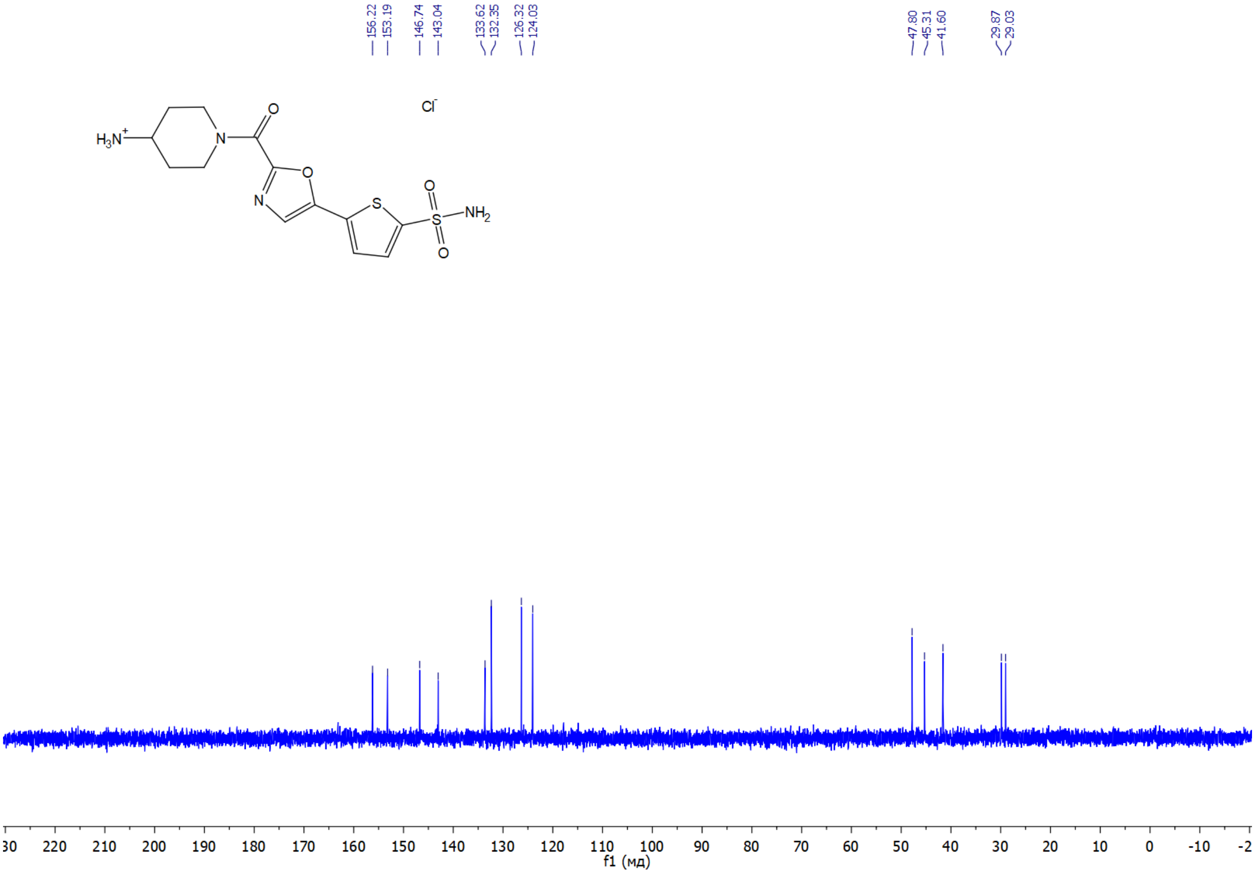

Copies of ^{1}H and ^{13}C spectra of compound **7d**

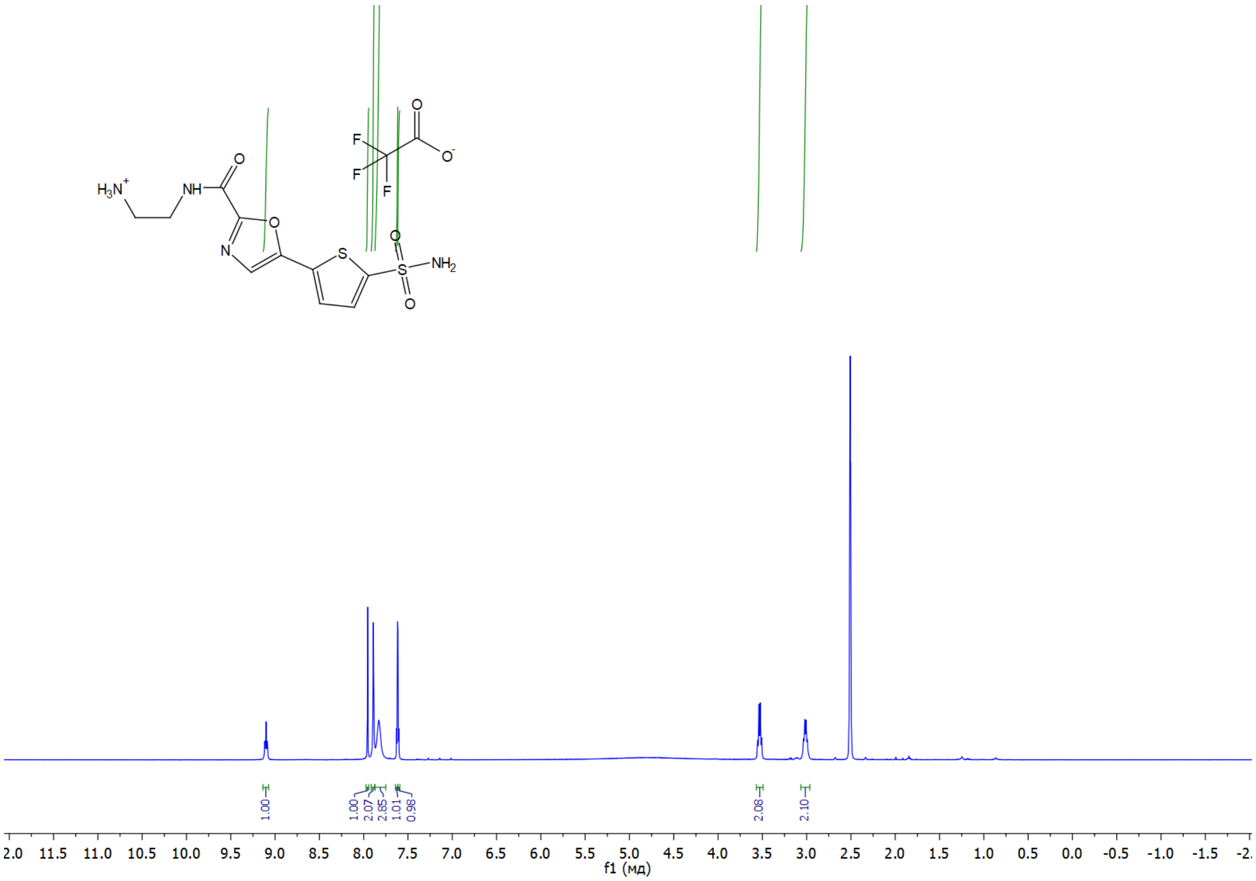

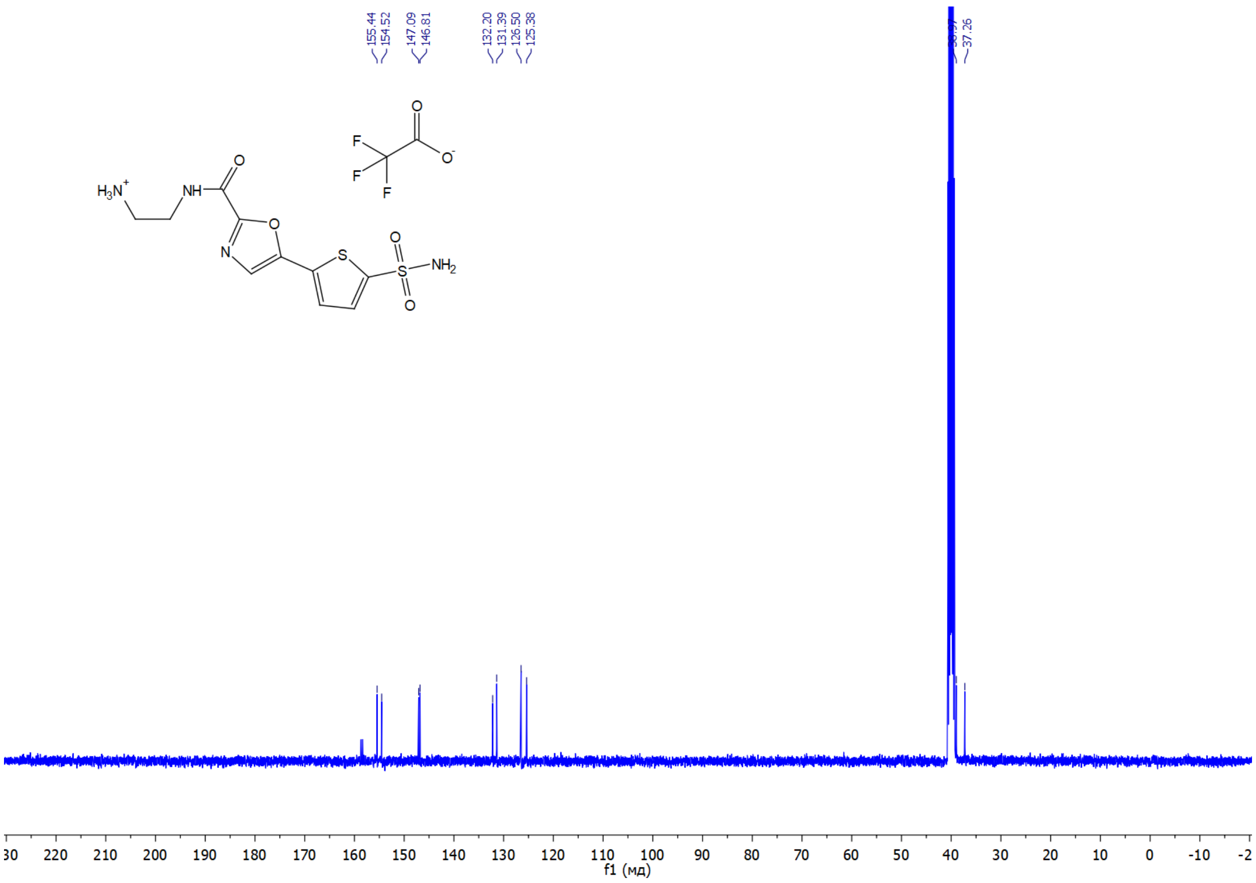

Copies of ^{1}H and ^{13}C spectra of compound **7e**

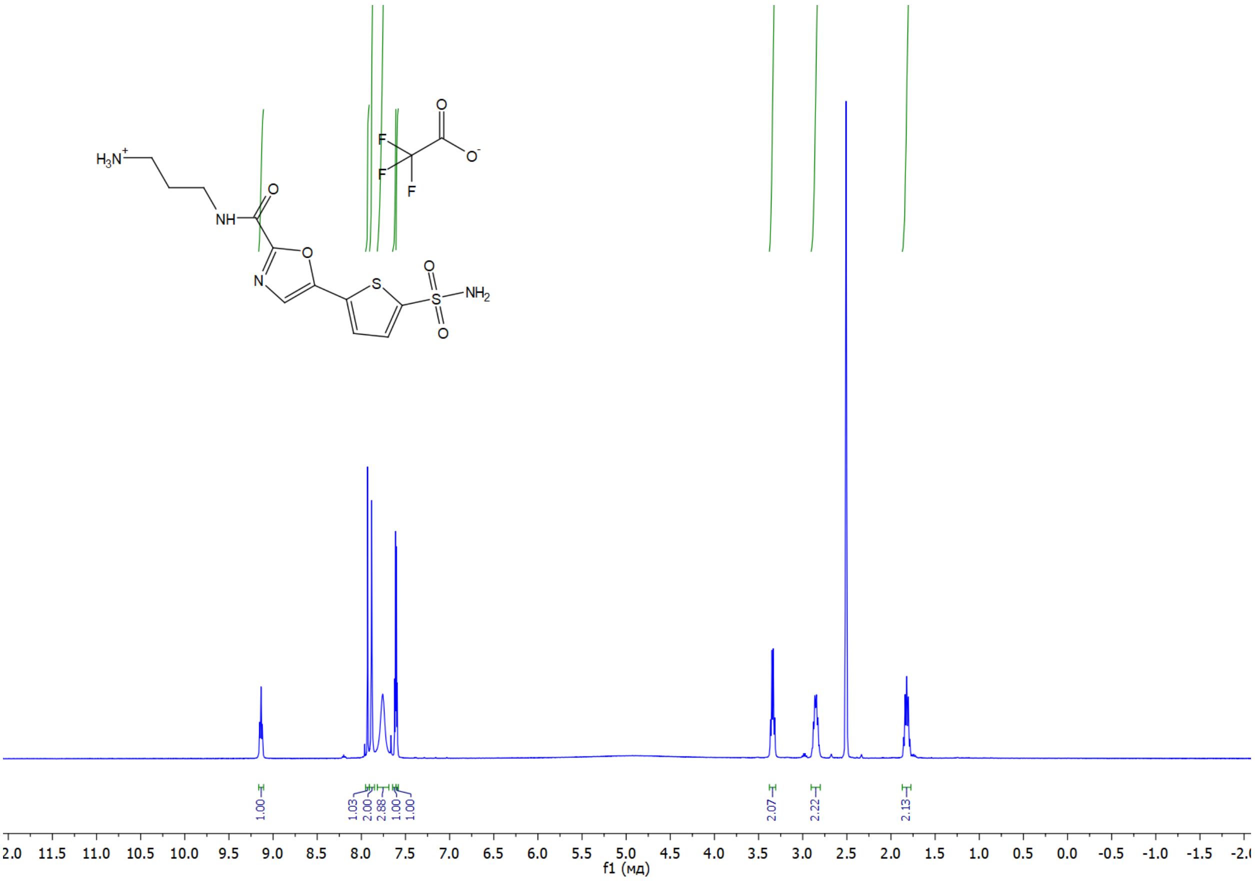

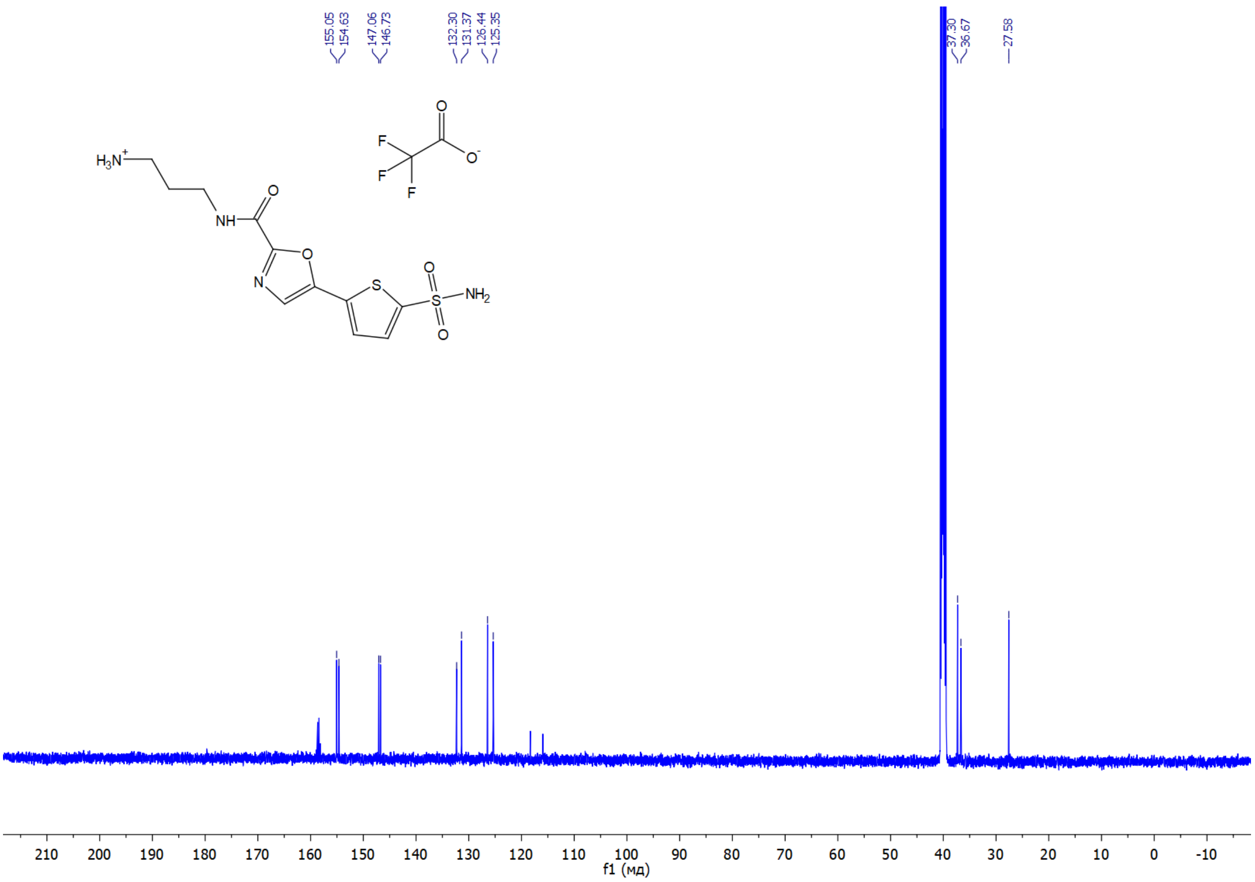

Carbonic anhydrase inhibition assay

An Applied Photophysics stopped-flow instrument has been used for assaying the CA catalyzed CO_2 hydration activity[1]. Phenol red (at a concentration of 0.2 mM) has been used as indicator, working at the absorbance maximum of 557 nm, with 20 mM Tris (pH 8.3) as buffer, and 20 mM Na_2SO_4 (for maintaining constant the ionic strength), following the initial rates of the CA-catalyzed CO_2 hydration reaction for a period of 10–100 s. The CO_2 concentrations ranged from 1.7 to 17 mM for the determination of the kinetic parameters and inhibition constants. For each inhibitor at least six traces of the initial 5–10% of the reaction have been used for determining the initial velocity. The uncatalyzed rates were determined in the same manner and subtracted from the total observed rates. Stock solutions of inhibitor (0.1 mM) were prepared in distilled-deionized water and dilutions up to 0.005 nM were done thereafter with the assay buffer. Inhibitor and enzyme solutions were preincubated together for 15 min at room temperature prior to assay, in order to allow for the formation of the E-I complex. The inhibition constants were obtained by non-linear least-squares methods using PRISM 3 and the Cheng-Prusoff equation, as reported earlier, and represent the mean from at least three different determinations. All CA isoforms were recombinant ones obtained in-house[2-5].

Docking and molecular dynamics studies

The crystal structure of hCA II (PDB code 2AW1[6]) was downloaded from the Protein Data Bank.[7] Protein-ligand complex was preprocessed using Schrodinger Protein PrepWizard.[8] Complexed ligand is removed from the active site and the protein structure was minimized using Schrodinger Prime module and OPLS3e force field at 310 K. Glide Grid program was used to prepare docking grid with included positional constraints. As a constraining factor interaction of sulfamide group with zinc ion in active site and hydrogen bond with Thr 200 residue was used. Grid spacing was set up as 0.375 Å. Grid box size was selected as 8 × 8 × 8 Å. Docking procedure was carried out with Schrodinger Glide program[9] using OPLS3e force field. Before docking, the receptor constraints were included, where needed, ligand features was marked to use. Energy window of 2 kcal/mol for conformer generation was chosen. Docked compounds subjected to 15 docking runs, with generation of 5000 poses per structure. Reference ligand geometry was used for binding pose control and best docked conformations were taken into account.

Intraocular pressure studies

New Zealand White young adult female rabbits from Envigo Laboratories (UK) were used in the experiments. The animals were housed under standard laboratory conditions of 12-hour dark-light

cycles and were provided with normal pellet diet with water ad libitum. Animals were handled in accordance with the statement of the Animals in Research Committee of the ARVO (Association for Research in Vision and Ophthalmology, Rockville, Maryland, USA) and all animal experiments were approved by the Finnish National Animal Experiment Board (Eläinkoelautakunta, ELLA). Commercial dorzolamide was used as reference drug (Trusopt®, dorzolamide 20 mg/mL, Santen Pharmaceutical Co., Ltd). The compound investigated (**7a**) was dissolved in PBS at 10 mg/mL concentration. The pH and osmolality (Auto-Osmometer Osmostat OM-6020, Kagaku Ca. Ltd.) of the eye drop solution was measured to ensure that the eye drops were close to neutrality and isotonicity. Before the start of the experiment, validation of animal model and some pre-experiments were done to habituate the animals for measuring and to get background information of the variability in individual animals, fellow eyes, days and the circadian rhythm. The drug was applied as single eye drop at volume of 25 µl to the left eye of the rabbit. The right eye was left untreated. The rabbits were held immobile for one minute after administration. Tonometer (iCare PRO, Icare Finland Ltd.) was used to measure intraocular pressure (IOP). In all experiments, the same eye was measured three times and the average values were used to calculate the value for the time point. The IOP of six normotensive rabbits were measured from both eyes before the administration of the molecule and at the timepoints 0.5, 1, 2, 3, 4, 5, 6, 7 and 8 h after treatment.

References

1. Khalifah RGJ. The carbon dioxide hydration activity of carbonic anhydrase. I. Stopflow kinetic studies on the native human isoenzymes B and C. Biol Chem. 1971;246:2561–2573.
2. Maresca A, Carta F, Vullo D, Supuran CT. Dithiocarbamates strongly inhibit the β-class carbonic anhydrases from *Mycobacterium tuberculosis*. J Enzyme Inhib Med Chem. 2013;28:407–411.
3. Ekinci D, Kurbanoglu NI, Salamci E, Senturk M, Supuran CT. Carbonic anhydrase inhibitors: inhibition of human and bovine isoenzymes by benzenesulphonamides, cyclitols and phenolic compounds. J Enzyme Inhib Med Chem. 2012;27:845–848.
4. Ekinci D, Karagoz L, Ekinci D, Senturk M, Supuran CT. Carbonic anhydrase inhibitors: *in vitro* inhibition of α isoforms (hCA I, hCA II, bCA III, hCA IV) by flavonoids. J Enzyme Inhib Med Chem. 2013;28:283–288.
5. Alp C, Maresca A, Alp NA, et al. Secondary/tertiary benzenesulfonamides with inhibitory action against the cytosolic human carbonic anhydrase isoforms I and II. J Enzyme Inhib Med Chem. 2013;28:294–298.

6. Di Fiore A, Pedone C, D'Ambrosio K, Scozzafava A, De Simone G, Supuran CT. Carbonic anhydrase inhibitors: valdecoxib binds to a different active site region of the human isoform II as compared to the structurally related cyclooxygenase II “selective” inhibitor celecoxib. Bioorg Med Chem Lett. 2006;16:437–442.

7. Berman HM, Westbrook J, Feng Z, et al. The protein data bank. Nucleic Acids Res. 2000;28:235–242.

8. Sastry GM, Adzhigirey M, Day T, Annabhimoju R, Sherman W. Protein and ligand preparation: parameters, protocols, and influence on virtual screening enrichments. J Comput Aid Mol Des. 2013;27:221–234.

9. Repasky MP, Shelley M, Friesner RA, Flexible ligand docking with Glide, Current protocols in bioinformatics. 2007; Chapter 8: Unit 8,12.
